# Chinese cross-culturally adapted patient-reported outcome measures (PROMs) for knee disorders: a systematic review and assessment using the Evaluating the Measurement of Patient-Reported Outcomes (EMPRO) instrument

**DOI:** 10.1186/s13018-022-03399-5

**Published:** 2022-11-24

**Authors:** James Reeves Mbori Ngwayi, Kenedy Uzoma Obie, Jie Tan, JianBiao Xu, Mujahid Alizada, Daniel Edward Porter

**Affiliations:** 1 School of Clinical Medicine, Tsinghua University, Zijing Apartment 21, Haidian District, Beijing, 100084 China; 2 School of Clinical Medicine, Central South University, Changsha, 410011 China; 3 Department of Orthopedics, Beijing Huaxin Hospital, Clinical Medicine School, Tsinghua University, Beijing, 100016 China; 4 Department of Neurosurgery, The Second Affiliated Hospital Of Fujian Medical University, Quanzhou, Fujian Province China

**Keywords:** Knee PROMs, Cross-cultural adaptation, EMPRO, Measurement properties

## Abstract

**Background:**

Knee patient-reported outcome measures (PROMs) are widely used in research in China, but there is limited evidence on the quality of cross-culturally adapted and original Chinese PROMs. We investigated Chinese language knee PROMs to provide evidence for clinicians on their quality and to guide PROM choices.

**Method:**

A systematic literature search of databases: PUBMED, CINAHL, EMBASE, and CNKI, using adequate search strings and a three-step screen process identified relevant studies. An independent standardized assessment of the selected studies based on the Evaluating the Measurement of Patient-Reported Outcomes (EMPRO) tool was performed. Inter-rater reliability was assessed using intraclass coefficients (ICC).

**Results:**

Thirty-three articles corresponding to 23 knee PROMs were evaluated with EMPRO global scores (100) ranging from 11.11 to 55.42. The attributes ‘reliability,’ ‘validity,’ and ‘cultural and language adaptation’ were significantly better evaluated compared to the attributes ‘responsiveness,’ ‘interpretability,’ and ‘burden’ (for all comparisons *p* < 0.0001). Moderate-to-excellent inter-rater agreement was observed with ICC values ranging from 0.538 to 0.934.

**Conclusion:**

We identified six PROMs with a minimum acceptable threshold (> 50/100). The osteoarthritis of knee and hip quality of life, the lower extremity function scale, and the Western Ontario Meniscal Evaluation tool ranked highest. Nevertheless, no single PROM had evidence encompassing all EMPRO attributes, necessitating further studies, especially on responsiveness, interpretability, and burden. We identified duplication of effort as shown by repeated translations of the same PROM; this inefficiency could be ameliorated by rapid approval of Chinese language PROMs documented on original PROM developers’ platforms.

**Supplementary Information:**

The online version contains supplementary material available at 10.1186/s13018-022-03399-5.

## Background

The output obtained from patient-reported outcome measures (PROMs), centered on patient perspectives and quality of life, has gained recognition as an important metric in clinical practice and research [1]. Total knee arthroplasty (TKA) PROMs are commonly used but studies have reported limited evidence for their psychometric properties and advise caution in PROM selection [2]. The use of poor-quality or unsuitable PROMs can introduce bias through unreliable effect estimates for these outcomes, leading to ethical concerns [3]. The need for quality has prompted several research groups to develop standards for the assessment of PROMs. These include the Streiner, the Evaluating the Measurement of Patient-Reported Outcomes (EMPRO), and Consensus-based Standards for the Selection of Health Measurement Instruments (COSMIN) [4–6]. EMPRO has the advantage of being semiquantitative, and has been used to assess shoulder-specific PROMs [7], while COSMIN has been used to assess total knee arthroplasty and elbow-specific PROMs [2, 8]; these studies focus on the evaluation of measurement properties of the original PROMs which are predominantly in English.

In mainland China, where the majority of outcome measures are translated into cross-cultural adaptations, evaluation of PROM quality is sparse. Such studies could serve as a basis for clinical PROM selection as they can provide readily available data to guide orthopedic researchers’ choice of PROM as well as identify areas requiring future research. The aim of this study was therefore to perform a systemic literature review to identify available Chinese language cross-cultural adapted PROMS, followed by an evaluation of available measurement properties based on EMPRO standards.

## Methods

### Identification of knee studies

A search was performed of the earliest records up to 22/08/2020 according to guidelines of the Preferred Reporting Items for Systematic Reviews and Meta-Analyses [9] (see PRISMA checklist). The following databases were selected: PubMed/MEDLINE, EMBASE (OVID), CINAHL (EBSCO), and CNKI (Chinese database). The search strings used were designed with MeSH terms and combinations of keywords based on previously described and documented strategies for PROM searches [3, 10, 11] (see Additional file 1), which were then tailored to the knee anatomical region and Chinese population. The publication languages for the articles were English and Chinese.

### Screening of articles and instruments

The screening was carried out as a three-step process (Titles, Abstract, and Full texts) and performed independently by two reviewers. Outputs were compared and a consensus was reached. After full-text screen and identification of suitable articles, we manually reviewed the in-article reference lists for potentially relevant articles missed during the electronic search.

Based on Population, Intervention, Comparison, Outcome (PICO) criteria, the following inclusion criteria were adopted: (1) cross-culturally adapted and translated knee PROMs tested in the Mainland Chinese population; (2) knee-specific PROMs evaluating interventions for knee disorders; and (3) PROMs restricted to the Chinese Mainland and written in simplified Chinese.

*Exclusion Criteria were* (1) PROMs written in traditional Chinese; (2) PROMs tested on populations out with Mainland China; and (3) articles not meeting inclusion criteria.

### Evaluating the Measurement of Patient-Reported Outcomes (EMPRO)

The EMPRO instrument consists of 8 attributes and 39 items, designed for quality assessment of PROMs: Conceptual and measurement model (items 1–7), Cultural and language Adaptations of the instrument (items 8–10), Reliability (items 11–18), Validity (items 19–24), Responsiveness (items 25–27), Interpretability (items 28–30), Burden (items 31–37), and Alternative modes of administration (items 38–39) [5].

Quantitative assessment for each item is via a 4-point Likert scale, graded from 4 (strongly agree) to 1 (strongly disagree). Alternative option boxes are ‘No information’ and ‘Not applicable.’ A short free-comments box is included for appraisers to document the rationale for item grading. The appraiser is required to provide an overall recommendation for the PROM use according to the following response scale: ‘Strongly recommended,’ ‘Recommended with provisos or alterations,’ ‘Would not recommend’ and ‘Not enough information.’

The EMPRO tool requires a license application via the portal www.bibliopro.org, which is free-to-use, and presently available in two languages (English and Spanish).

### Standardized and systematic evaluation

Following the systematic review, the specific instruments under investigation were identified from full-text articles. For PROMs of non-Chinese origin, the original development publication was also retrieved (see Additional file 2). A standardized assessment of the adequacy of their measurement properties was undertaken using the EMPRO tool. According to recommendations from the designers, two reviewers (both clinicians with a background in PROMs research) performed the assessment. Both had completed the online EMPRO training webinar (https://www.isoqol.org/category/webinar/page/3/). The assessment was carried out in two phases. The first phase consisted of each reviewer independently scoring article(s) supporting each cross-culturally adapted Knee PROM for methodological attributes, as well as the article describing the original design of the PROM for the conceptual and measurement model assessment. The second phase which followed a consensus method recommended by the EMPRO designers involved discussions between reviewers on discrepancies to obtain a common score for each item [5]. Reviewers were based on two continents and did not converse on scoring until the discussion phase.

After the first phase of independent scoring by reviewers, an agreement between them was assessed by using a two-way, random, single unit, absolute agreement intraclass correlation coefficients ICC [12]. The degree of reviewer agreement was categorized based on Cicchetti (1994): ICC < 0.40 poor; 0.40–0.59 moderate; 0.60–0.74 good; and 0.75–1.00 excellent [13].

### Scoring and analysis

Scoring of the methodological attributes was calculated based on developers’ instructions; https://www.isoqol.org/?s=Empro. Specifically, attribute-specific scores are obtained by calculating the response mean of the applicable items when at least 50% of them are rated; and items check-marked with the option ‘no information’ are assigned a score of 1 representing the lowest possible item score. The response means for each attribute are then linearly transformed to a range of 0–100 (worst to best). Global scores (based on metric properties) are only calculated when at least three attributes can be scored. Attributes without information are imputed zero. Panoramic assessment (which includes all culture/language versions of the instrument) involves conceptual model, reliability, validity, responsiveness, and interpretability, while culture/language-specific evaluation involves conceptual model, reliability, validity, responsiveness and interpretability, and cross-cultural adaptation. The EMPRO domains are elaborate and strictly designed to avoid ceiling effects, making a score of 100 (maximum score) difficult to obtain; thus, a score of 50 (half of the maximum) is considered to be an acceptable threshold [5, 14]. We applied this minimum threshold in our result analysis.

Analysis and graphics were designed with Microsoft Excel 2003 (Microsoft, Redmond, WA, USA). Differences in scores between EMPRO attributes were compared using the nonparametric Mann–Whitney test. Inter-rater reliability was performed using SPSS^®^ Version 20.0 software (SPSS Inc., Chicago, IL, USA).

## Results

### Identification of studies and screening

**Fig. 1:**
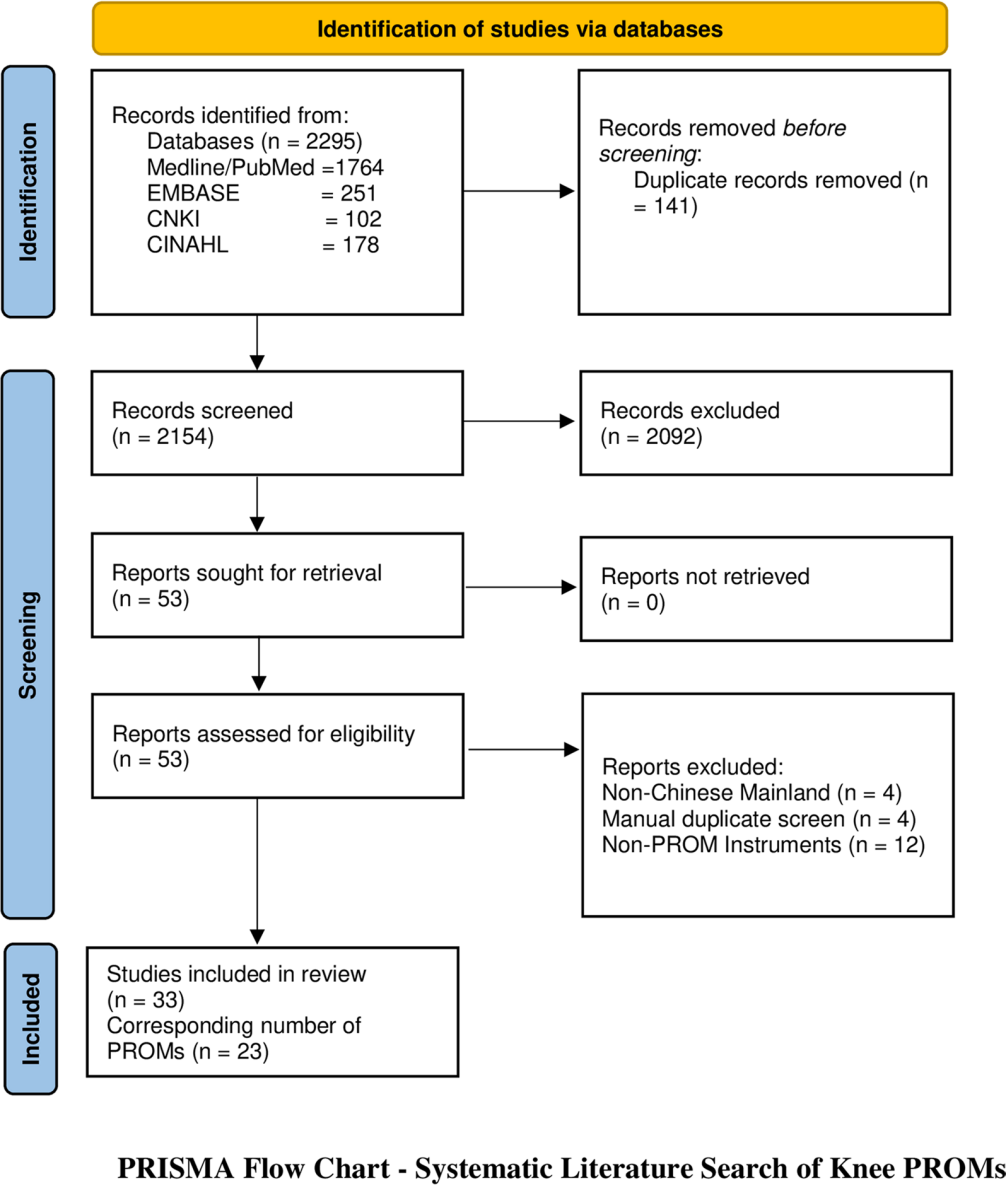
PRISMA flowchart—systematic literature search of knee PROMs

The literature search resulted in an initial pool of 2295 studies. Articles retrieved per database were as follows: PubMed/MEDLINE 1764 (76.9%), EMBASE 251 (10.9%), CINAHL 102 (4.4%), and CNKI 178 (7.8%). After the removal of duplicates, the number of abstracts for further screening was 2145. Articles were discarded successively following the three-phase process previously described in the Methods section. A manual search was performed on the references of full-text articles and no new articles on PROMs were identified. Fifty-three full-text articles were screened in detail using the predefined inclusion criteria resulting in the exclusion of 20 studies; 4 evaluated non-Chinese mainland patient population, 12 were non-PROM instruments, and 4 duplicates were screened manually. The PRISMA chart of the review process is illustrated in Fig. 1.

A total of 33 articles were retained for evaluation consisting of 23 separate Chinese language PROMs used in the evaluation of knee disorders. Twenty-two PROMs were cross-cultural adaptations and modifications of preexisting tools, while one PROM was originally developed in China. Two articles evaluated two PROMs simultaneously, increasing the total number of PROM psychometric assessments to 35. Table 1 describes the general characteristics of each, including dimensionality and scale for each PROM.

**Table 1:** General characteristics of Chinese cross–culturally adapted knee PROMs

Instrument	Author (year)	Population for evaluation	Number of items and dimensions	Scale
Forgotten joint score (FJS)	Cao S et al. (2017)	Post-TKA for Degenerative KOA Mean age 68.1	12 items	Standardized on a scale from 0 (high degree of awareness) to 100 (low degree of awareness)
Forgotten joint score (FJS)	Wang Z et al. (2020)	Post-unicompartmental TKA Mean age: 61.8	12 items	Standardized on a scale from 0 (high degree of awareness) to 100 (low degree of awareness)
University of California at Los Angeles activity score for arthroplasty and arthroscopy (UCLA)	Cao S et al. (2017)	TKA and Arthroscopy for KOA, cartilage defect or meniscal tear Mean age: Arthroscopy: 48.8, Arthroplasty: 69.4	1 item	1 (worst) to 10 (best)
Oxford knee score (OKS)	Chen C et al. (2020)	KOA for TKA Mean age: 63.7	12 items	0 (worst outcome) to 48 (best outcome)
Oxford knee score (OKS)	Lin K et al. (2017)	Unilateral TKA for KOA Mean age 67.6		
Oxford knee score (OKS)	Wu H et al. (2020)	Unilateral TKA for KOA Mean age: N/A		
Oxford knee score (OKS)	Li YJ et al. (2019)	KOA Mean age: 64.1		
Oxford knee score (OKS)	Chen S et al. (2018)	KOA Mean age 59.3		
Anterior Cruciate ligament return to sport after injury scale (ACL-RSI)	Chen T et al. (2017)	Post-ACLR Mean age: 26.6	12 items: Emotions (5), Confidence in performance (5), Risk appraisal (2)	11-point Numeric Rating Scale (NRS) from 0 (worst) to 100 (best)
Anterior Cruciate ligament return to sport after injury scale (ACL-RSI)	Jia ZY et al. (2018)	Post-ACLR Mean age: 34	12 items: Emotions (5), Confidence in performance (5), Risk appraisal (2)	
Tegner Activity Score	Huang H et al. (2016)	Pre-ACLR and Post-ACLR Mean age: 29.5	1 item	0 (worst) to 10 (best)
Knee outcome survey activities of daily living scale (KOS ADL)	Jia ZY et al. (2016)	Knee disorders Mean age: 44.4	14 items: Symptoms (6), Functionality (8)	Standardized 0 (worst) to 100 (best)
International knee documentation committee subjective knee form (IKDC)	Jia ZY et al. (2018)	Knee disorders: degenerative changes, ligamentous injuries, meniscus injuries, patellofemoral pain, or patellar dislocation Mean age: 49.4	18-item: Symptoms (7), Function (1), Sports activity (10)	Standardized 0 (best) to 100 (worst)
Activity and participation questionnaire(APQ)	Chen C et al. (2020)	KOA Mean age: 63.7	8 items	0 (worst) to 32 (best)
Western Ontario and McMaster University Osteoarthritis index scale (WOMAC)	Symonds T et al. (2015)	KOA Mean age: 59.1	24 items: Pain (5), Stiffness (2 s), Physical function (17)	Standardized on a scale from 0 (best) to 100 (worst)
Western Ontario meniscal evaluation tool (WOMET)	Tong WW et al. (2016)	Meniscal injury—Post-arthroscopic repair Mean age: 41.2	16 items: Physical symptoms (9), Sports/reaction/ work/lifestyle (4), Emotions (3)	0 (best) to 1600 (worst)
Lysholm knee score	Wang W et al. (2016)	ACL injury Mean age: 25.9	8 items	0 (worst) to 100 (best)
The osteoarthritis of knee and hip quality of life (OAKHQOL)	Wang W et al. (2016)	Osteoarthritis for both hip and knee arthroplasty Mean age 63	5 Domains: Physical activities (16), Mental health (13), Pain (4), Social support (4), social activities (3). Others are three independent items related to relationships, sexual activity and professional life	Standardized on a scale from 0 (worst quality of life) to 100 (best quality of life)
Lower extremity function scale (LEFS)	Xu L et al. (2020)	KOA—Arthroplasty Mean age: 65.1	20 items	0 (worst) to 80 (best)
Lower extremity function scale (LEFS)	Lu Nan (2015)	KOA Mean age: 63.4	20 items	0 (worst) to 80 (best)
Frenchay Activities Index (FAI)	Zhang TJ et al. (2014)	Post-TKR Mean age: 59.4	15 items: Domestic (5), Leisure/work (5), Outdoors (5)	15 (worst) to 60 (best)
Frenchay Activities Index (FAI)	Lian HR et al. (2014)	Post-TKR Mean age: 59.4		
Knee injury and osteoarthritis outcome score (KOOS)	Zhang QH et al. (2019)	ACL injury Mean age: 44.86	42 items: Pain (9), Symptoms (7), Activities of daily life function (17), Sports and Recreation Function (5), Quality of Life (4)	Standardized on a scale from 0 (worst) to 100 (best) for each domain
Knee injury and osteoarthritis outcome score (KOOS)	Sheng WJ (2011)	Cruciate ligament injury, meniscal tears, mixed injury Mean age: 46.7		
Knee injury and osteoarthritis outcome score (KOOS)	Wang Y et al. (2015)	ACL injury, meniscal injury, mixed injury, sprains, tibial plateau injury, KOA, Other knee injuries Mean age: 47.58		
Knee injury and osteoarthritis outcome score (KOOS)	Chen S et al. (2018)	KOA Mean age: 59.3		
International physical activity questionnaire (IPAQ)	Gao D et al. (2020)	Post-TKR Mean age: 60.22	7 items	http:// www.ipaq.ki.se Scoring Instructions
International physical activity questionnaire (IPAQ)	Lan PW et al. (2013)	Post-TKR Age > 50		
Knee self-efficacy scale (K-SES)	Zhao H (2015)	Post-ACLR Mean Age: 30.5	22 items: Daily activities (7), Sports and Leisure activities (5), Physical activities (6), Your knee function in the future (4)	Items are scored on an 11-point scale (0 = not certain at all and 10 = very certain)
Musculoskeletal quality of health (MSK HQ)	Pu Y (2019)	Post-unilateral TKA Age range: 45–79	14 items	0 (worst) to 56 (best)
Japanese knee osteoarthritis measure (JKOM)	Xu SY et al. (2014)	KOA Mean age: 63.35	25 items: Pain/stiffness, (7), Activities of daily living (6), Movement / role / participation / health perception (12)	Each item is scored from 0–4 and the pain item is scored on a 10 point VAS scale
Knee osteoarthritis traditional Chinese medicine syndrome evaluation scale (KOA-TCM-SES)	Huang SM (2017)	KOA Mean age: 60.6	11 items	0 (best) to 99 (worst)
Modified Western Ontario and McMaster University osteoarthritis index scale (Modified WOMAC)	Shen ZD. et al. (2019)	KOA Mean age: 59	22 items: Pain (5), Stiffness (2), Function (12), Quality of Life (3)	0 (best) to 88 (worst)
Intermittent and constant osteoarthritis pain (ICOAP)	Zhang C et al. (2017)	KOA Mean age: 63.77	11 items: Constant pain (5), Intermittent pain (6)	0 (worst) to 44 (best)
Tampa scale for Kinesiophobia (TSK-11)	Cai L et al. (2019)	Post-unilateral TKA Mean age 63.1	11 items	11 (best) to 44 (worst)

*Key*, KOA knee osteoarthritis, *ACL*, anterior cruciate ligament, *ACLR*, anterior cruciate ligament repair, *TKA*, total knee arthroplasty, *TKR*, total knee replacement, *N/A*, not available. For table references see Additional file 3

Data from both of the Forgotten Joint Score (FJS) articles could be aggregated, reducing the number of separate EMPRO evaluations from 35 to 34. Following precedent in other studies [7, 15], the global score for each domain was transformed into a five-point scale (denoted: − / + / ++ / +++ / +++). + : EMPRO score < 25; ++ : EMPRO score 25–49; +++ : EMPRO score 50–74; ++++ : EMPRO score 75–100; − : EMPRO score not applicable or not calculable according to designer instructions (see Additional file 4).

**Fig. 2:**
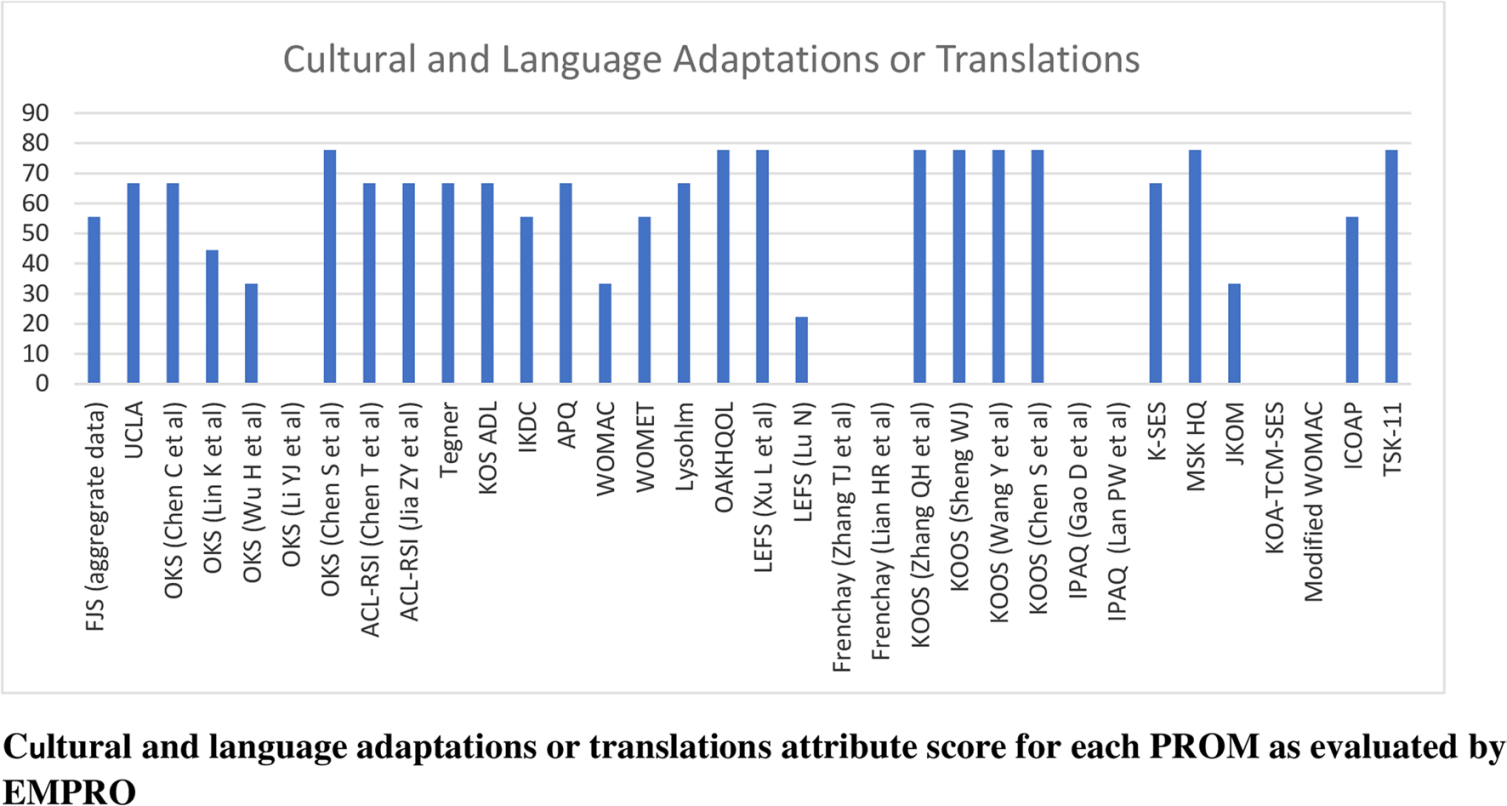
Cultural and language adaptations or translations attribute score for each PROM as evaluated by EMPRO

**Fig. 3:**
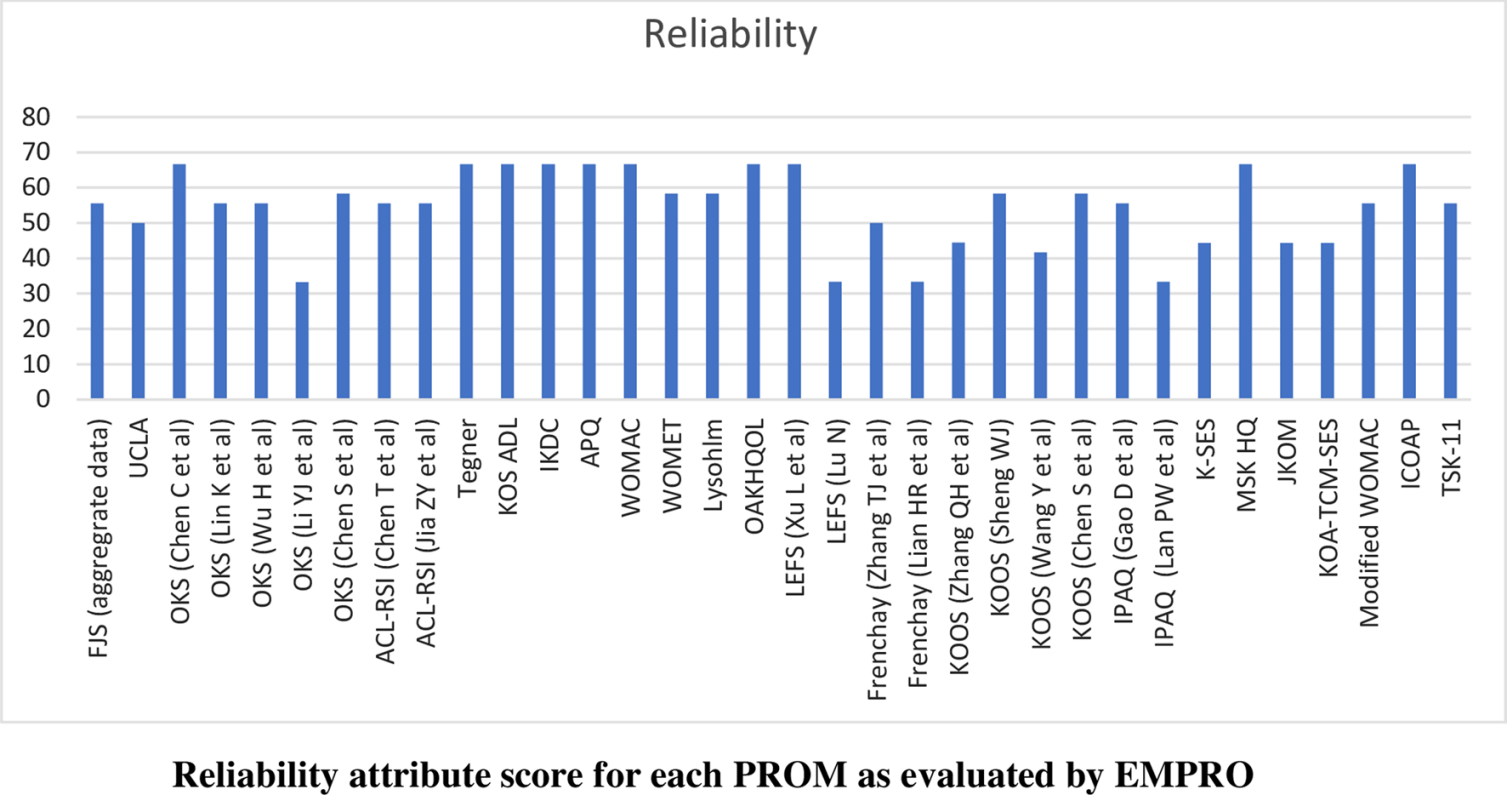
Reliability attribute score for each PROM as evaluated by EMPRO

**Fig. 4:**
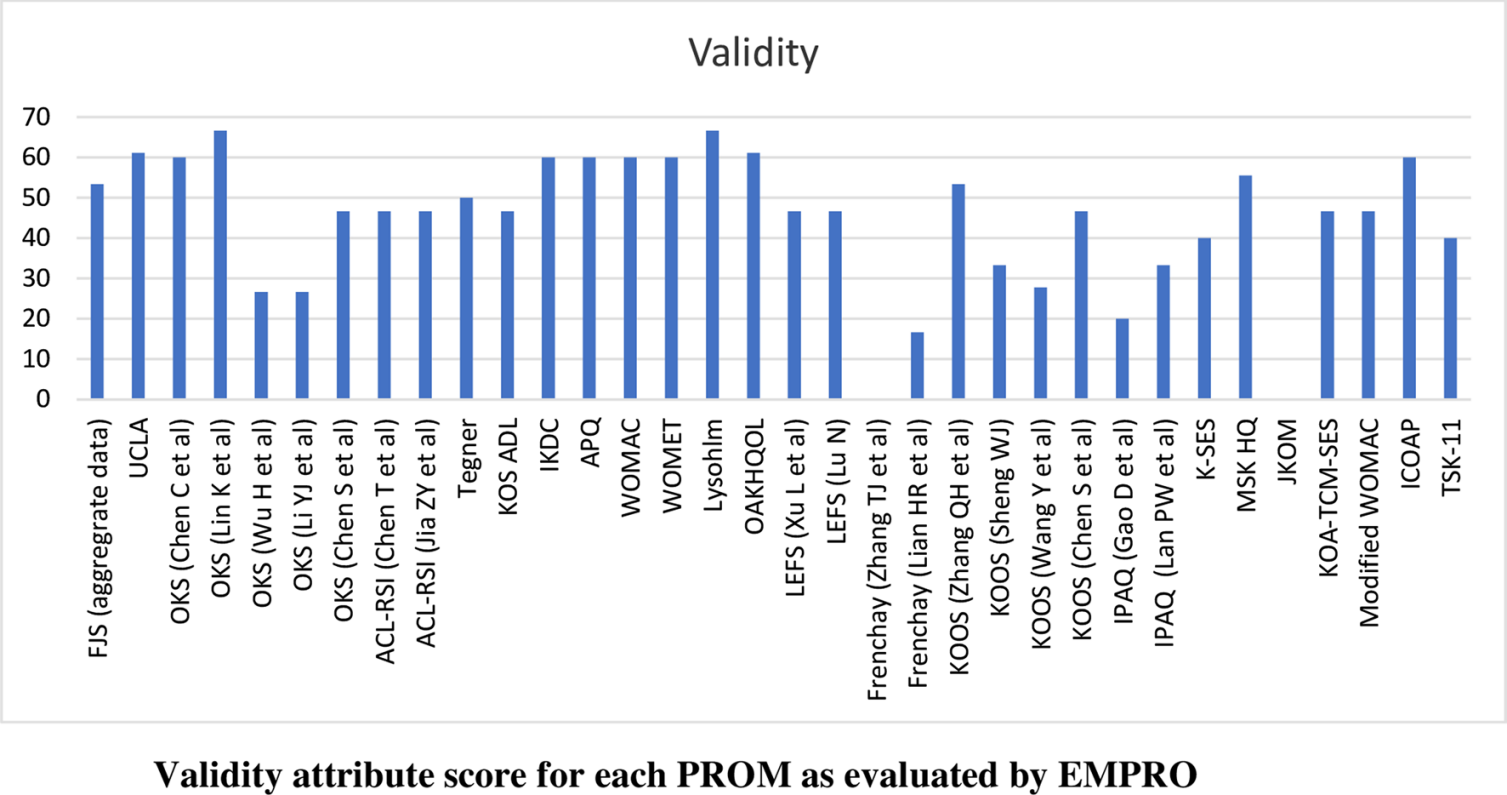
Validity attribute score for each PROM as evaluated by EMPRO

**Fig. 5:**
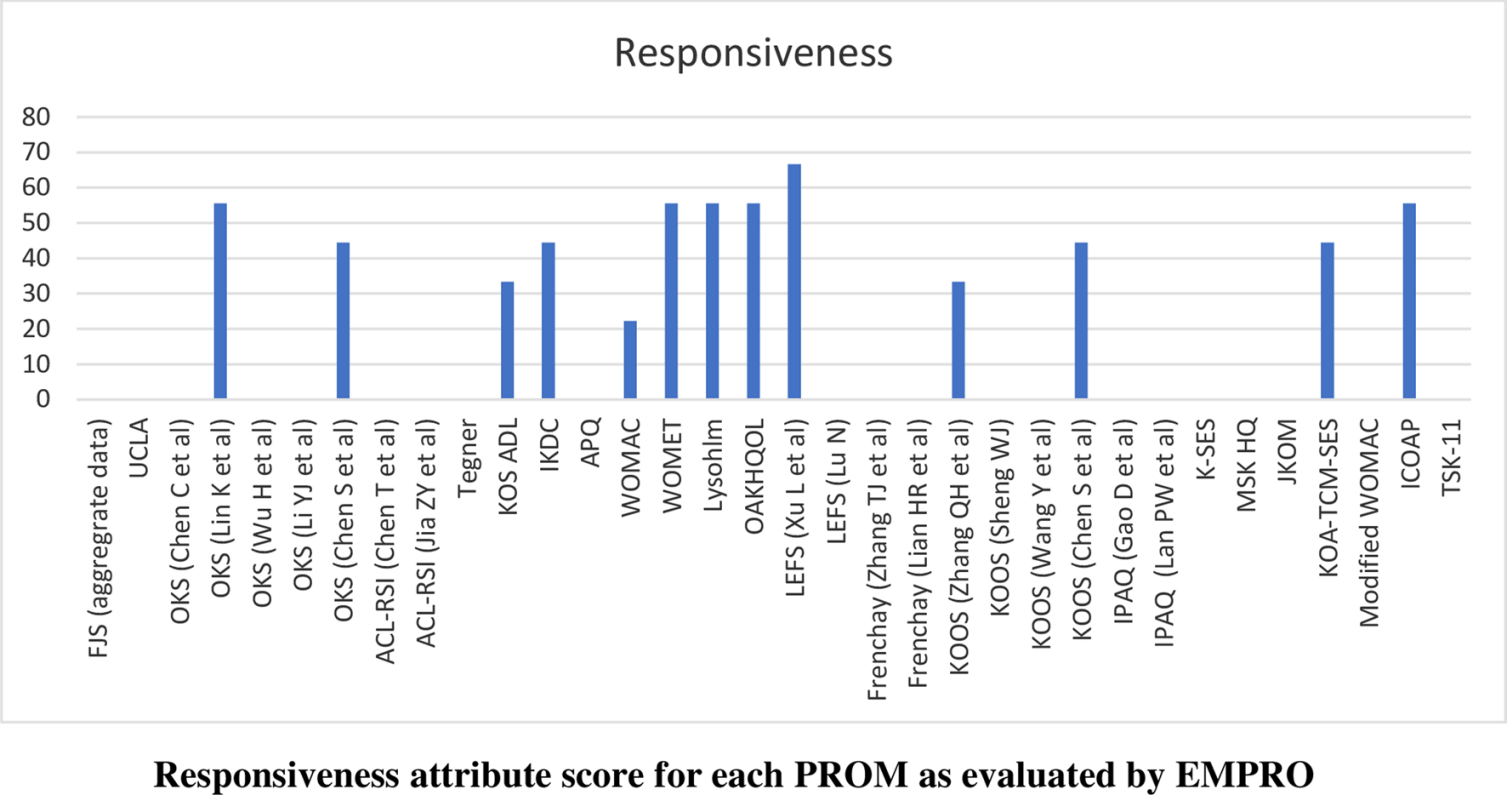
Responsiveness attribute score for each PROM as evaluated by EMPRO

**Fig. 6:**
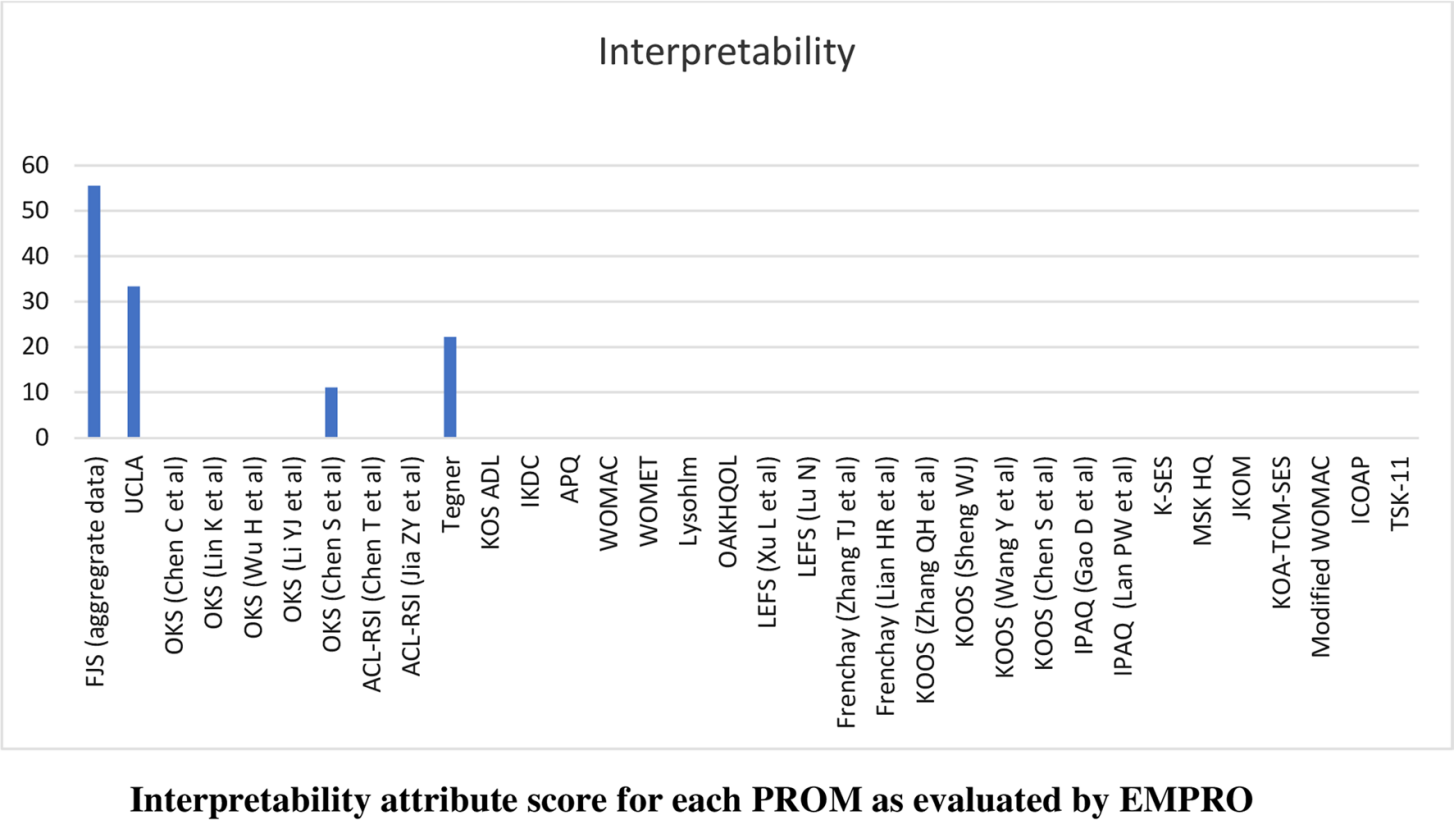
Interpretability attribute score for each PROM as evaluated by EMPRO

**Fig. 7:**
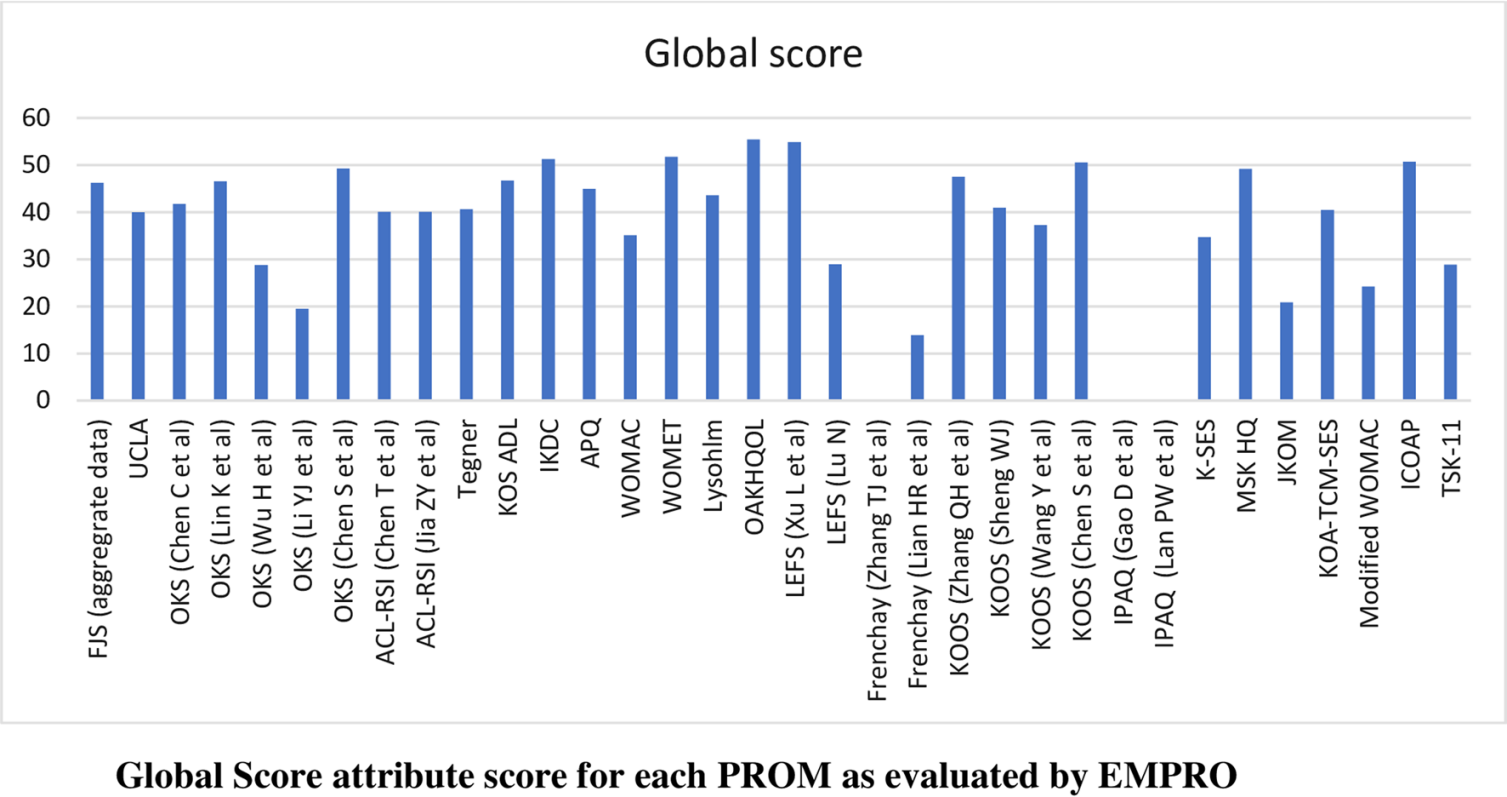
Global score attribute score for each PROM as evaluated by EMPRO

Scores for the conceptual equivalence and measurement model attributes were obtained from the originator articles and could not be calculated for two PROMs (International Physical Activity Questionnaire and the Tampa Scale for Kinesiophobia) due to the absence of more than 50% of rateable items) and was not applicable for non-culturally adapted Knee Osteoarthritis Traditional Chinese Medicine Syndrome Evaluation Scale (KOA-TCM-SES). Altogether six studies could not be scored for the cultural and language adaptation attribute and two for the validity attribute. The proportion of PROMs with attributes reaching ‘acceptability’ (score > 50/100) was 68% for conceptual and measurement model, 65% for cultural and language adaptation (Fig. 2), 74% for reliability (Fig. 3), 38% for validity (Fig. 4), 6% for responsiveness (Fig. 5), 3% for interpretability (Fig. 6), and 0% for burden. Domains ‘cultural and language adaptation,’ ‘reliability,’ and ‘validity’ scored significantly higher compared to ‘responsiveness,’ ‘interpretability,’ and ‘burden’ (all *p* < 0.0001). For the 31 Chinese language PROMs studies which had three or more rateable attributes, panoramic global scores ranged from 11.11 to 55.42. Six studies reached an overall ‘acceptable’ threshold; the International Knee Documentation Committee Subjective Knee Form (IKDC), the Western Ontario Meniscal Evaluation Tool (WOMET), the Osteoarthritis of Knee and Hip Quality of Life (OAKHQOL), the Lower Extremity Function Scale (LEFS), the Knee Injury and Osteoarthritis Outcome Score (KOOS) and the Intermittent and Constant Osteoarthritis Pain (ICOAP) (range 50.56–55.43) (Fig. 7).

Agreement between the two reviewers based on intraclass correlation coefficient (ICC) was moderate for evaluations of the University of California at Los Angeles Activity Score for Arthroplasty and Arthroscopy (UCLA) (0.538), good for the Tegner Activity Score and one of the Frenchay Activities Index (FAI) versions (0.687 and 0.736, respectively), and excellent for the remaining articles evaluated (0.753–0.934).

## Discussion

Searches of the Chinese literature for evaluations of PROM quality yielded no published research in musculoskeletal or other clinical disciplines with which to compare our data. In the English language literature, an independent assessment of psychometric qualities of knee instruments in 2010 recommended the Cincinnati Knee Rating System, KOOS, and the Lysholm Knee Score for anterior cruciate ligament (ACL) injuries, the Kujala Anterior Knee Pain Scale for anterior knee pain, the International Knee Documentation Committee (IKDC) Subjective Knee Form, KOOS, and Lysholm Knee Score for focal chondral defects, the WOMET for meniscal injuries, and the KOOS for osteoarthritis [16]. A subsequent systematic review of PROMs in Total Knee Arthroplasty used COSMIN guidelines to assess 32 instruments, among which 12 PROMs were cross-culturally adapted [2]. The authors found limited psychometric evidence to support their widespread use. Only three PROMs had four or more properties demonstrating positive evidence out of the nine psychometric attributes analyzed: the Work, Osteoarthritis or Joint Replacement Questionnaire (WORQ), the Oxford Knee Score (OKS) and the Western Ontario and McMaster Universities Arthritis Index (WOMAC). They recommended WORQ as demonstrating the strongest evidence for use in TKA patients. Commonly used PROMs for osteoarthritis research include the OKS, KOOS, and WOMAC [17]. Among instruments recommended by these three research groups, the Cincinnati Knee Rating System, the Kujala Anterior Knee Pain Scale, and WORQ have not been evaluated in mainland Chinese populations.

Using EMPRO which has considerable overlap with COSMIN, we found similar deficiencies in cross-culturally adapted Chinese PROMs. We evaluated and scored articles documenting the measurement properties of 23 distinct PROMs used in knee disorders and associated therapeutic interventions. The Chinese cross-cultural adaptations were used on patients with a range of knee disorders including osteoarthritis, meniscal, ligament, and soft tissue injuries, similar to the patient populations for which these were originally designed.

Six PROMs (OAKHQOL, LEFS, WOMET, IKDC, ICOAP, and KOOS) had panoramic global scores above the threshold for acceptability; however, no PROM achieved a score higher than 55.43; these were ‘recommended with provisos’ by our raters.

We found multiple studies on the cross-cultural adaptation of some PROMs such as the OKS which had five supporting articles containing separate and independent translated versions. Among separate studies on the KOOS, three were based on a Singapore Chinese version (the official version recommended on the developer’s Web site), but were validated on patients with disparate population characteristics, making data aggregation unfeasible. Only data from two Forgotten Joint Score articles could be aggregated as they referenced a single Chinese language version and patient populations were similar.

We identified the attributes of reliability and validity to be generally well-evidenced. Cultural and language adaptation also scored highly, although differential item functioning and harmonization were often under-documented. Harmonization ensures conceptual equivalence between the source and target language versions and between all translations in order to guarantee the safe aggregation of data from different language versions [18].

Interpretability refers to the degree to which one can assign qualitative meaning to the quantitative score of the instrument. In clinical practice, a PROM that does not reflect or predict clinical epidemiological effects may be uninterpretable [19]. This attribute was the least well evaluated.

PROMs are by definition outcome scores; however, responsiveness to intervention was evaluated in only 13 studies since the majority were cross-sectional.

Although burden is not a psychometric property, implementing PROMs into clinical practice requires effective delivery and therefore minimal effort for patients, clinicians, and administrators. Data on this attribute were omitted by most authors.

Deficient attributes highlighted in Figs. 2, 3, 4, 5, 6, 7 (Additional file 4) identify areas of further research to potentially improve the overall quality of each PROM. The results of the present study are therefore not final but are subject to change following new published evidence on psychometric properties.

Limitations of the present study include reliance on a single instrument (EMPRO), with subjective attribute assessment; although bias was reduced by using two experienced raters who demonstrated moderate to excellent inter-rater reliability. We chose EMPRO due to easier visualization of relative PROM quality which may be of particular help for clinicians without detailed metric science knowledge; as opposed to nonquantitative evaluation such as COSMIN. However, the properties evaluated by EMPRO overlap with COSMIN and other assessment tools [2, 3]. Some autonomous regions in China use PROMs in traditional Chinese text [20], so the scope of the present study was purposefully restricted to mainland China with PROMs written in simplified Chinese; additional studies in other regions could provide a holistic picture of Knee PROMs used in China and in the Chinese diaspora.

## Conclusion

The present study evaluated Chinese cross-cultural adaptation and translated studies and identified six PROMs, suitable for a range of knee conditions that attained minimum threshold for acceptability. We identified occasional duplication of effort and suboptimal documentation of language version sources. We recommend clinicians to use Chinese PROMs approved and documented on developers’ Web sites to encourage efficiency and unanimity. Although cross-cultural adaptation, reliability, and validity were generally assessed as higher-quality attributes, responsiveness, interpretability, and burden were less so; thus, effort should be directed toward evaluating and providing evidence for deficient attributes. Several high-quality PROMs have not yet been translated and evaluated in mainland Chinese populations.

## Supplementary Information

**Additional file 1**. PubMed/MEDLINE Search filter.

**Additional file 2**. References for Original developed PROMs.

**Additional file 3**. Table References.

**Additional file 4**. Global Scores for each PROM.

## Data Availability

The datasets used and/or analyzed during the current study are available from the corresponding author upon reasonable request.

## Abbreviations

PROMs: Patient-reported outcome measures
EMPRO: Evaluating the Measurement of Patient-Reported Outcomes
ICC: Intraclass coefficients
OAKHQOL: The osteoarthritis of knee and hip quality of life
LEFS: The Lower Extremity Function Scale
WOMET: The Western Ontario Meniscal Evaluation Tool
TKA: Total knee arthroplasty
COSMIN: Consensus-based standards for the selection of health measurement instruments
PICO: Population, Intervention, Comparison, Outcome
FJS: Forgotten joint score
KOA-TCM-SES: Knee osteoarthritis traditional Chinese medicine syndrome evaluation scale
IKDC: The International Knee Documentation Committee Subjective Knee Form
KOOS: Knee injury and osteoarthritis outcome score
ICOAP: Intermittent and constant osteoarthritis pain
UCLA: University of California at Los Angeles Activity score for arthroplasty and arthroscopy
FAI: Frenchay Activities Index
ACL: Anterior cruciate ligament
WORQ: Work, osteoarthritis or joint replacement questionnaire
OKS: Oxford knee score
WOMAC: Western Ontario and McMaster Universities Arthritis Index
