# Supplementary material for: Chinese cross-culturally adapted patient-reported outcome measures (PROMs) for knee disorders: a systematic review and assessment using the Evaluating the Measurement of Patient-Reported Outcomes (EMPRO) instrument
Source: J Orthop Surg Res. 2022 Nov 24;17:508. doi: 10.1186/s13018-022-03399-5 (PMC9694593; doi:10.1186/s13018-022-03399-5)
Supplement: Supplementary file 1 — Additional file 1. PubMed/MEDLINE Search filter. [file 13018_2022_3399_MOESM1_ESM.docx]

**Supplementary Material : PubMed/MEDLINE filter. Psychometric properties of Knee-specific PROMs in the Chinese population**

**#1: Construct search**

(HR-PRO OR HRPRO OR HRQL OR HRQoL OR QL OR QoL OR quality of life OR (health index* OR health indices or health profile*) OR health status OR ((patient or self OR carer OR proxy) adj (appraisal* or appraised OR report OR reported OR reporting OR rated OR rating* OR based OR assessed OR assessment*)) OR (disability or function OR functional OR functions OR subjective OR utility OR utilities OR wellbeing or well being OR priorit* OR waiting))

**#2: population search**

(Knee[tiab] OR Knee [MeSH]

**#3: instrument search**

(index OR indices OR instrument OR instruments OR measure OR measures OR questionnaire* OR profile OR profiles OR scale OR scales OR score OR scores OR status OR survey OR surveys)

**#4 geographical search**

(China or Chinese or (Chinese adj version) or (Chinese adj validation) or (Chinese adj translation) or (Crosscultural adj adaptation) or (Cross-cultural adj validation)

**#4: 1 AND #2 AND #3 AND filter for measurement properties**

Validation Studies[pt] OR Comparative Study[pt] OR “psychometrics”[MeSH] OR psychometr*[tiab] OR clinimetr*[tw] OR clinometr*[tw] OR “outcome assessment (health care)”[MeSH] OR outcome assessment[tiab] OR outcome measure*[tw] OR “observer variation”[MeSH] OR observer variation[tiab] OR “Health Status Indicators”[Mesh] OR “reproducibility of results”[MeSH] OR reproducib*[tiab] OR “discriminant analysis”[MeSH] OR reliab*[tiab] OR unreliab*[tiab] OR valid*[tiab] OR coefficient[tiab] OR homogeneity[tiab] OR homogeneous[tiab] OR “internal consistency”[tiab] OR (cronbach*[tiab] AND (alpha[tiab] OR alphas[tiab])) OR (item[tiab] AND (correlation*[tiab] OR selection*[tiab] OR reduction*[tiab])) OR agreement[tiab] OR precision[tiab] OR imprecision[tiab] OR “precise values”[tiab] OR test–retest[tiab] OR (test[tiab] AND retest[tiab]) OR (reliab*[tiab] AND (test[tiab] OR retest[tiab])) OR stability[tiab] OR interrater[tiab] OR inter-rater[tiab] OR intrarater[tiab] OR intra-rater[tiab] OR intertester[tiab] OR inter-tester[tiab] OR intratester[tiab] OR intra-tester[tiab] OR interobserver[tiab] OR inter-observer[tiab] OR intraobserver[tiab] OR intra-observer[tiab] OR intertechnician[tiab] OR inter-technician[tiab] OR intratechnician[tiab] OR intra-technician[tiab] OR interexaminer[tiab] OR inter-examiner[tiab] OR intraexaminer[tiab] OR intra-examiner[tiab] OR interassay[tiab] OR inter-assay[tiab] OR intraassay[tiab] OR intra-assay[tiab] OR interindividual[tiab] OR inter-individual[tiab] OR intraindividual[tiab] OR intra-individual[tiab] OR interparticipant[tiab] OR inter-participant[tiab] OR intraparticipant[tiab] OR intra-participant[tiab] OR kappa[tiab] OR kappa’s[tiab] OR kappas[tiab] OR repeatab*[tiab] OR ((replicab*[tiab] OR repeated[tiab]) AND (measure[tiab] OR measures[tiab] OR findings[tiab] OR result[tiab] OR results[tiab] OR test[tiab] OR tests[tiab])) OR generaliza*[tiab] OR generalisa*[tiab] OR concordance[tiab] OR (intraclass[tiab] AND correlation*[tiab]) OR discriminative[tiab] OR “known group”[tiab] OR factor analysis[tiab] OR factor analyses[tiab] OR dimension*[tiab] OR subscale*[tiab] OR (multitrait[tiab] AND scaling[tiab] AND (analysis[tiab] OR analyses[tiab])) OR item discriminant[tiab] OR interscale correlation*[tiab] OR error[tiab] OR errors[tiab] OR “individual variability”[tiab] OR (variability[tiab] AND (analysis[tiab] OR values[tiab])) OR (uncertainty[tiab] AND (measurement[tiab] OR measuring[tiab])) OR “standard error of measurement”[tiab] OR sensitiv*[tiab] OR responsive*[tiab] OR ((minimal[tiab] OR minimally[tiab] OR clinical[tiab] OR clinically[tiab]) AND (important[tiab] OR significant[tiab] OR detectable[tiab]) AND (change[tiab] OR difference[tiab])) OR (small*[tiab] AND (real[tiab] OR detectable[tiab]) AND (change[tiab] OR difference[tiab])) OR meaningful change[tiab] OR “ceiling effect”[tiab] OR “floor effect”[tiab] OR “Item response model”[tiab] OR IRT[tiab] OR Rasch[tiab] OR “Differential item functioning”[tiab] OR DIF[tiab] OR “computer adaptive testing”[tiab] OR “item bank”[tiab] OR “cross-cultural equivalence”[tiab])

**#5: #4 limit to English and Chinese**

**#6: #5 limit to Humans**
