# Supplementary material for: Chinese cross-culturally adapted patient-reported outcome measures (PROMs) for knee disorders: a systematic review and assessment using the Evaluating the Measurement of Patient-Reported Outcomes (EMPRO) instrument
Source: J Orthop Surg Res. 2022 Nov 24;17:508. doi: 10.1186/s13018-022-03399-5 (PMC9694593; doi:10.1186/s13018-022-03399-5)
Supplement: Supplementary file 2 — Additional file 2. References for Original developed PROMs. [file 13018_2022_3399_MOESM2_ESM.docx]

**Supplementary Material – References for original development PROM publication used for evaluation**

1. Miller RP, Kori S, Todd D. The Tampa Scale: a measure of kinesiophobia. Clin J Pain. 1991;7(1):51–52
2. Behrend H, Giesinger K, Giesinger JM, Kuster MS. The "forgotten joint" as the ultimate goal in joint arthroplasty: validation of a new patient-reported outcome measure. J Arthroplasty. 2012 Mar;27(3):430-436.e1. doi: 10.1016/j.arth.2011.06.035.
3. Amstutz HC, Thomas BJ, Jinnah R, Kim W, Grogan T, Yale C. Treatment of primary osteoarthritis of the hip. A comparison of total joint and surface replacement arthroplasty. J Bone Joint Surg Am. 1984 Feb;66(2):228-41.
4. Dawson J, Fitzpatrick R, Murray D, Carr A. Questionnaire on the perceptions of patients about total knee replacement. J Bone Joint Surg Br. 1998 Jan;80(1):63-9. doi: 10.1302/0301-620x.80b1.7859.
5. Dawson J, Beard DJ, McKibbin H, Harris K, Jenkinson C, Price AJ. Development of a patient-reported outcome measure of activity and participation (the OKS-APQ) to supplement the Oxford knee score. Bone Joint J. 2014 Mar;96-B(3):332-8. doi: 10.1302/0301-620X.96B3.32845.
6. Webster KE, Feller JA, Lambros C. Development and preliminary validation of a scale to measure the psychological impact of returning to sport following anterior cruciate ligament reconstruction surgery. Phys Ther Sport. 2008 Feb;9(1):9-15. doi: 10.1016/j.ptsp.2007.09.003.
7. Tegner Y, Lysholm J. Rating systems in the evaluation of knee ligament injuries. Clin Orthop Relat Res. 1985 Sep;(198):43-9.
8. Irrgang JJ, Snyder-Mackler L, Wainner RS, Fu FH, Harner CD. Development of a patient-reported measure of function of the knee. J Bone Joint Surg Am. 1998 Aug;80(8):1132-45. doi: 10.2106/00004623-199808000-00006.
9. Irrgang JJ, Anderson AF, Boland AL, et al. Development and Validation of the International Knee Documentation Committee Subjective Knee Form. *The American Journal of Sports Medicine*. 2001;29(5):600-613. doi:[10.1177/03635465010290051301](https://doi.org/10.1177/03635465010290051301)
10. Bellamy N, Buchanan WW. A preliminary evaluation of the dimensionality and clinical importance of pain and disability in osteoarthritis of the hip and knee. Clin Rheumatol. 1986 Jun;5(2):231-41. doi: 10.1007/BF02032362.
11. Kirkley A, Griffin S, Whelan D. The development and validation of a quality of life-measurement tool for patients with meniscal pathology: the Western Ontario Meniscal Evaluation Tool (WOMET). Clin J Sport Med. 2007 Sep;17(5):349-56. doi: 10.1097/JSM.0b013e31814c3e15.
12. Lysholm J, Gillquist J. Evaluation of knee ligament surgery results with special emphasis on use of a scoring scale. Am J Sports Med. 1982 May-Jun;10(3):150-4. doi: 10.1177/036354658201000306.
13. Rat AC, Pouchot J, Coste J, Baumann C, Spitz E, Retel-Rude N, Baumann M, Le Quintrec JS, Dumont-Fischer D, Guillemin F; Groupe Qualité de Vie en Rhumatologie. Development and testing of a specific quality-of-life questionnaire for knee and hip osteoarthritis: OAKHQOL (OsteoArthritis of Knee Hip Quality Of Life). Joint Bone Spine. 2006 Dec;73(6):697-704. doi: 10.1016/j.jbspin.2006.01.027.
14. Binkley JM, Stratford PW, Lott SA, Riddle DL. The Lower Extremity Functional Scale (LEFS): scale development, measurement properties, and clinical application. North American Orthopaedic Rehabilitation Research Network. Phys Ther. 1999 Apr;79(4):371-83.
15. Hawker GA, Davis AM, French MR, et al. Development and preliminary psychometric testing of a new OA pain measure--an OARSI/OMERACT initiative. *Osteoarthritis Cartilage*. 2008;16(4):409-414. doi:10.1016/j.joca.2007.12.015
16. Roos EM, Roos HP, Lohmander LS, Ekdahl C, Beynnon BD. Knee Injury and Osteoarthritis Outcome Score (KOOS)--development of a self-administered outcome measure. J Orthop Sports Phys Ther. 1998 Aug;28(2):88-96. doi: 10.2519/jospt.1998.28.2.88.
17. Craig CL, Marshall AL, Sjöström M, Bauman AE, Booth ML, Ainsworth BE, Pratt M, Ekelund U, Yngve A, Sallis JF, Oja P. International physical activity questionnaire: 12-country reliability and validity. Med Sci Sports Exerc. 2003 Aug;35(8):1381-95. doi: 10.1249/01.MSS.0000078924.61453.FB.
18. Holbrook M, Skilbeck CE. An activities index for use with stroke patients. Age Ageing. 1983 May;12(2):166-70. doi: 10.1093/ageing/12.2.166.
19. Akai M, Doi T, Fujino K, Iwaya T, Kurosawa H, Nasu T. An outcome measure for Japanese people with knee osteoarthritis. J Rheumatol. 2005 Aug;32(8):1524-32.
20. Hill JC, Kang S, Benedetto E*, et al*Development and initial cohort validation of the Arthritis Research UK Musculoskeletal Health Questionnaire (MSK-HQ) for use across musculoskeletal care pathways *BMJ Open*2016;**6:**e012331. doi: 10.1136/bmjopen-2016-012331
21. Thomeé P, Währborg P, Börjesson M, Thomeé R, Eriksson BI, Karlsson J. A new instrument for measuring self-efficacy in patients with an anterior cruciate ligament injury. Scand J Med Sci Sports. 2006 Jun;16(3):181-7. doi: 10.1111/j.1600-0838.2005.00472.x.
22. 柯询，谢利民，于潼，张振南，刘杨. 膝骨关节炎中医证候患者报告结局评价量表的研制[J]. Int J Trad Chin Med，2017，Vol. 39，(No.1)：8-12.
23. 沈正东， 于慧敏，王俊婷，师国洋，孙焱. 改良版西安大略和麦克马斯特大学骨关节炎 指数量表在膝骨关节炎中的应用 [J]. Natl Med J China， 2019， Vol. 99(No. 7)：537-541.
