# Supplementary material for: Chinese cross-culturally adapted patient-reported outcome measures (PROMs) for knee disorders: a systematic review and assessment using the Evaluating the Measurement of Patient-Reported Outcomes (EMPRO) instrument
Source: J Orthop Surg Res. 2022 Nov 24;17:508. doi: 10.1186/s13018-022-03399-5 (PMC9694593; doi:10.1186/s13018-022-03399-5)
Supplement: Supplementary file 3 — Additional file 3. Table References. [file 13018_2022_3399_MOESM3_ESM.docx]

**Supplementary Material - Table 1 References**

1. Cao S, Liu N, Han W, et al. Simplified Chinese version of the Forgotten Joint Score (FJS) for patients who underwent joint arthroplasty: cross-cultural adaptation and validation. J Orthop Surg Res. 2017;12(1):6. Published 2017 Jan 14. doi:10.1186/s13018-016-0508-5
2. Wang Z, Deng W, Shao H, et al. Forgotten Joint Score Thresholds for Forgotten Joint Status and Patient Satisfaction after Unicompartmental Knee Arthroplasty in Chinese Patients. J Arthroplasty. 2020 Oct;35(10):2825-2829. doi:10.1016/j.arth.2020.05.010. Epub 2020 May 11. PMID: 32482475.
3. Cao S, Liu N, Li L ,et al. Simplified Chinese Version of University of California at Los Angeles Activity Score for Arthroplasty and Arthroscopy: Cross-Cultural Adaptation and Validation. J Arthroplasty. 2017 Sep;32(9):2706-2711. doi: 10.1016/j.arth.2017.03.057. Epub 2017 Apr 13. PMID: 28483213.
4. Chen C, Wang W, Wu H, et al. Cross-cultural translation and validation of the Chinese Oxford Knee Score and the Activity and Participation Questionnaire. J Orthop Surg (Hong Kong). 2020 Jan-Apr;28(2):2309499020910668. doi: 10.1177/2309499020910668. PMID: 32301381.
5. Lin K, Bao L, Wang J, et al. Validation of the Chinese (Mandarin) Version of the Oxford Knee Score in Patients with Knee Osteoarthritis. Clin Orthop Relat Res. 2017 Dec;475(12):2992-3004. doi: 10.1007/s11999-017-5495-2. Epub 2017 Sep 7. PMID: 28884273; PMCID: PMC5670067.
6. 吴昊, 王渭君，宁仁德, 王双利. 中文版牛津膝关节功能评估量表在中国大陆人群中的应用 [J]. 蚌埠医学院学报,2020,45(3):371- 4.
7. 李阳杰，孙奇峰，邓传超，胡逍然，尹文哲.牛津膝关节评分量表应用于国内东北地区膝骨关节炎的信度和效度分析 [J]. 实用骨科杂志，2019，25(10)936-40.
8. 陈墅，曹嘉，蔡筑韵，李章吉，杜杰，顾召华，周琦, 钱齐荣. 中文版 KOOS 和 OKS 在评估膝骨关节炎患者多模式非手术治疗中的对比研究 [J]. Chinese Journal of Bone and Joint Surgery，Aug.2018，Vol.11(8)：600-605.
9. Chen T, Zhang P, Li Y, et al. Translation, cultural adaptation and validation of simplified Chinese version of the anterior cruciate ligament return to sport after injury (ACL-RSI) scale. PLoS One. 2017 Aug 17;12(8):e0183095. doi: 10.1371/journal.pone.0183095. PMID: 28817645; PMCID: PMC5560729.
10. Jia ZY, Cui J, Wang W, et al. Translation and validation of the simplified Chinese version of the anterior cruciate ligament-return to sport after injury (ACL-RSI). Knee Surg Sports Traumatol Arthrosc. 2018 Oct;26(10):2997-3003. doi: 10.1007/s00167-018-4850-5. Epub 2018 Feb 5. PMID: 29404654.
11. Huang H, Zhang D, Jiang Y, et al. Translation, Validation and Cross-Cultural Adaptation of a Simplified-Chinese Version of the Tegner Activity Score in Chinese Patients with Anterior Cruciate Ligament Injury. PLoS One. 2016;11(5):e0155463. Published 2016 May 17. doi:10.1371/journal.pone.0155463
12. Jia ZY, Wang W, Nian XW, et al. Cross-cultural Adaptation and Validation of the Simplified Chinese Version of the Knee Outcome Survey Activities of Daily Living Scale. Arthroscopy. 2016 Oct;32(10):2009-2016. doi: 10.1016/j.arthro.2016.01.068. Epub 2016 Apr 28. PMID: 27132769.
13. Jia ZY, Zhang C, Zou Y, et al. Translation and validation of the Simplified Chinese version of International Knee Documentation Committee Subjective Knee Form. Arch Orthop Trauma Surg. 2018 Oct;138(10):1433-1441. doi: 10.1007/s00402-018-2973-2. Epub 2018 Jun 5. PMID: 29869690.
14. Chen C, Wang W, Wu H, et al. Cross-cultural translation and validation of the Chinese Oxford Knee Score and the Activity and Participation Questionnaire. J Orthop Surg (Hong Kong). 2020 Jan-Apr;28(2):2309499020910668. doi: 10.1177/2309499020910668. PMID: 32301381.
15. Symonds T, Hughes B, Liao S, et al. Validation of the Chinese Western Ontario and McMaster Universities Osteoarthritis Index in Patients From Mainland China With Osteoarthritis of the Knee. *Arthritis Care Res (Hoboken)*. 2015;67(11):1553-1560. doi:10.1002/acr.22631
16. Tong WW, Wang W, Xu WD. Development of a Chinese version of the Western Ontario Meniscal Evaluation Tool: cross-cultural adaptation and psychometric evaluation. *J Orthop Surg Res*. 2016;11(1):90. Published 2016 Aug 15. doi:10.1186/s13018-016-0424-8
17. Wang W, Liu L, Chang X, et al. Cross-cultural translation of the Lysholm knee score in Chinese and its validation in patients with anterior cruciate ligament injury. *BMC Musculoskelet Disord*. 2016;17(1):436. Published 2016 Oct 19. doi:10.1186/s12891-016-1283-5
18. Wang W, He CR, Zheng W, et al. Development of a valid simplified Chinese version of the Osteoarthritis of Knee and Hip Quality of Life (OAKHQOL) in patients with knee or hip osteoarthritis. *J Eval Clin Pract*. 2016;22(1):53-61. doi:10.1111/jep.12431
19. Xu L, Wang C, Zhang C, Feng X, et al. Cross-cultural adaption and validation of simplified Chinese version of the lower extremity function scale in patients with knee osteoarthritis. *Clin Rheumatol*. 2020;39(10):3041-3048. doi:10.1007/s10067-020-05077-5
20. 陆南南. 下肢功能评价量表(LEFS)在膝关节骨性关节炎患者中应用 的信度和效度 [D]； 首都体育学院， 2015.
21. 张挺久，张东，曾凡伟，庾明，王枰稀，王进. 老年患者行全膝关节置换术后活动量评估量表的信度和效度分析 [J]. Sichuan Medical Journal， 2014， 35(12)： 1576-7.
22. 连海荣，姜丽英，罗建成，王波. Frenchay活动量表评估老年患者行全膝关节置换术后活动量的信度和效度分析 [J]. 陕西医学杂志，2014 年8月，43(8)：961-2.
23. Zhang QH, Du SX, Zheng GZ, et al. Reliability, Validity, and Responsiveness of the Chinese Version of the Knee Injury and Osteoarthritis Outcome Score (KOOS) in Patients with Anterior Cruciate Ligament Reconstruction in Mainland China. Reliabilität, Validität und Änderungssensitivität der chinesischen Version des „Knee Injury and Osteoarthritis Outcome Score“ (KOOS) bei Patienten mit Rekonstruktion des vorderen Kreuzbandes in Festlandchina. *Z Orthop Unfall*. 2019;157(1):42-47. doi:10.1055/a-0621-9504
24. SHENG W J. Validation and reliability of the Chinese Version of the Knee Injury and Osteoarthritis Outcome Score for patients with injury [D]； Shantou University， 2011/04.
25. 王影，王钢， 许敬丽， 赖剑强， 曹生鲁，汪祎然. 中文版膝关节损伤及骨性关节炎转归评分的 信度和效度评价 [J]. Guangdong Medical Journal， Aug. 2015， 36(16).
26. 陈墅，曹嘉，蔡筑韵，李章吉，杜杰，顾召华，周琦, 钱齐荣. 中文版 KOOS 和 OKS 在评估膝骨关节炎患者多模式非手术治疗中的对比研究 [J]. Chinese Journal of Bone and Joint Surgery， Aug.2018， Vol.11(8)：600-605.
27. 兰平文，沈彬，杨静，周宗科，康鹏德，裴福兴. 国际活动量问卷用于全膝关节置换术后患者活动量评估的信度和效度研究 [J]. Chinese Journal of Rehabilitation Medicine， 2013， 28(8)： 743-6.
28. 高张，张晓瑞，潘治军，李耀章，白马恒，刘增亮，张亮，马建兵. IPAQ 长问卷表在全膝关节置换术后 患者活动量应用的信度与效度分析研究 [J]. Journal of Practical Orthopaedics， Mar.2020， Vol.26(No.3).
29. 赵辉. 中文版膝关节自我效能量表的信效度研究 [J]. Journal of Nursing Science， Nov. 2015 Vol.30 (No.22)
30. 蒲颖. 肌肉骨骼健康问卷的汉化以及在全膝关节置换术 [D]； 海南医学院， 2019.
31. 徐守宇，姚新苗，吴燕，张丽梅，李高权，刘雪云，黑泽尚，赤居正美. 汉化版日本膝关节骨关节炎功能评估量表的信度研究[J]. Chinese Journal of Rehabilitation Medicine， 2014， 29(8)： 723-5.
32. 黄松珉.膝骨关节炎中医证候PRO量表信度、效度及反应度评价 [D]；北京中医学大学，2017.
33. 沈正东， 于慧敏，王俊婷，师国洋，孙焱. 改良版西安大略和麦克马斯特大学骨关节炎 指数量表在膝骨关节炎中的应用 [J]. Natl Med J China， 2019， Vol. 99(No. 7)：537-541.
34. Zhang C, Liu DH, Qu YL, et al. Transcultural adaptation and validation of the Chinese version of the intermittent and constant osteoarthritis pain (ICOAP) measure in patients with knee osteoarthritis. *Osteoarthritis Cartilage*. 2017;25(4):506-512. doi:10.1016/j.joca.2016.11.012
35. Cai L, Liu Y, Woby SR, et al. Cross-Cultural Adaptation, Reliability, and Validity of the Chinese Version of the Tampa Scale for Kinesiophobia-11 Among Patients Who Have Undergone Total Knee Arthroplasty. *J Arthroplasty*. 2019;34(6):1116-1121. doi:10.1016/j.arth.2019.01.076
