# Supplementary material for: Chinese cross-culturally adapted patient-reported outcome measures (PROMs) for knee disorders: a systematic review and assessment using the Evaluating the Measurement of Patient-Reported Outcomes (EMPRO) instrument
Source: J Orthop Surg Res. 2022 Nov 24;17:508. doi: 10.1186/s13018-022-03399-5 (PMC9694593; doi:10.1186/s13018-022-03399-5)
Supplement: Supplementary file 4 — Additional file 4. Global Scores for each PROM. [file 13018_2022_3399_MOESM4_ESM.docx]

**Supplementary Material . Attribute and global scores for each PROM on a five-point scale according to methodology described in the Results section of the manuscript**

| **PROM** | **Concept and measu-rement model** | **Cultural and Language Adaptations** | **Reliab-ility** | **Vali-dity** | **Respon-siveness** | **Interp-**  **retability** | **Bur-den** | **Total Score /100** |
| --- | --- | --- | --- | --- | --- | --- | --- | --- |
| **FJS (aggregate data)** | +++ | +++ | +++ | +++ | - | +++ | + | 46.19 |
| **UCLA** | ++ | +++ | +++ | +++ | - | ++ | - | 39.95 |
| **OKS (Chen C et al)** | +++ | +++ | +++ | +++ | - | - | + | 41.75 |
| **OKS (Lin K et al)** | +++ | ++ | +++ | +++ | +++ | - | - | 46.57 |
| **OKS (Wu H et al)** | +++ | ++ | +++ | ++ | - | - | - | 28.79 |
| **OKS (Li YJ et al)** | +++ | - | ++ | ++ | - | - | + | 19.52 |
| **OKS (Chen S et al)** | +++ | ++++ | +++ | ++ | ++ | + | - | 49.24 |
| **ACL-RSI (Chen T et al)** | +++ | +++ | +++ | ++ | - | - | + | 40.06 |
| **ACL-RSI (Jia ZY et al)** | +++ | +++ | +++ | ++ | - | - | - | 40.06 |
| **Tegner** | ++ | +++ | +++ | +++ | - | + | - | 40.61 |
| **KOS ADL** | +++ | +++ | +++ | ++ | ++ | - | - | 46.67 |
| **IKDC** | ++++ | +++ | +++ | +++ | ++ | - | - | 51.28 |
| **APQ** | ++++ | +++ | +++ | +++ | - | - | - | 44.93 |
| **WOMAC** | ++ | ++ | +++ | +++ | + | - | - | 35.14 |
| **WOMET** | ++++ | +++ | +++ | +++ | +++ | - | - | 51.73 |
| **Lysholm** | + | +++ | +++ | +++ | +++ | - | + | 43.58 |
| **OAKHQOL** | +++ | ++++ | +++ | +++ | +++ | - | ++ | 55.43 |
| **LEFS (Xu L et al)** | +++ | ++++ | +++ | ++ | +++ | - | + | 54.87 |
| **LEFS (Lu N)** | +++ | + | ++ | ++ | - | - | ++ | 28.94 |
| **Frenchay (Zhang TJ et al)** | ++ | - | +++ | - | - | - | - | N/A |
| **Frenchay (Lian HR et al)** | ++ | - | ++ | + | - | - | - | 13.89 |
| **KOOS (Zhang QH et al)** | ++++ | ++++ | ++ | +++ | ++ | - | - | 47.51 |
| **KOOS (Sheng WJ)** | ++++ | ++++ | +++ | ++ | - | - | + | 40.94 |
| **KOOS (Wang Y et al)** | ++++ | ++++ | ++ | ++ | - | - | + | 37.24 |
| **KOOS (Chen S et al)** | ++++ | ++++ | +++ | ++ | ++ | - | - | 50.57 |
| **IPAQ (Gao D et al)** | - | - | +++ | + | - | - | - | N/A |
| **IPAQ (Lan PW et al)** | - | - | ++ | ++ | - | - | - | N/A |
| **K-SES** | +++ | +++ | ++ | ++ | - | - | + | 34.70 |
| **MSK HQ** | ++++ | ++++ | +++ | ++ | - | - | ++ | 49.21 |
| **JKOM** | ++ | ++ | ++ | - | - | - | + | 20.89 |
| **KOA-TCM-SES** | +++ | - | ++ | ++ | ++ | - | + | 40.43 |
| **Modified WOMAC** | ++ | - | +++ | ++ | - | - | - | 24.18 |
| **ICOAP** | +++ | +++ | +++ | +++ | +++ | - | + | 50.75 |
| **TSK-11** | - | ++++ | +++ | ++ | - | - | + | 28.89 |
